# Viral etiology, seasonality and severity of hospitalized patients with severe acute respiratory infections in the Eastern Mediterranean Region, 2007–2014

**DOI:** 10.1371/journal.pone.0180954

**Published:** 2017-07-13

**Authors:** Katherine C. Horton, Erica L. Dueger, Amr Kandeel, Mohamed Abdallat, Amani El-Kholy, Salah Al-Awaidy, Abdul Hakim Kohlani, Hanaa Amer, Abel Latif El-Khal, Mayar Said, Brent House, Guillermo Pimentel, Maha Talaat

**Affiliations:** 1 Global Disease Detection Center, U.S. Centers for Disease Control and Prevention, Cairo, Egypt; 2 Global Disease Detection and Response Program, U.S. Naval Medical Research Unit, No.3, Cairo, Egypt; 3 Global Disease Detection Branch, U.S. Centers for Disease Control and Prevention, Atlanta, Georgia, United States of America; 4 Preventive Sector, Ministry of Health and Population, Cairo, Egypt; 5 Communicable Disease Department, Ministry of Health, Amman, Jordan; 6 Clinical Pathology Department, Cairo University Hospitals, Cairo, Egypt; 7 Communicable Disease Department, Ministry of Health, Muscat, Oman; 8 Communicable Disease Department, Ministry of Health, Sana’a, Yemen; 9 Clinical Pathology Department, Ain Shams University, Cairo, Egypt; 10 Section of Medicine, Hamad Medical Corporation, Doha, Qatar; Public Health Agency of Canada, CANADA

## Abstract

**Introduction:**

Little is known about the role of viral respiratory pathogens in the etiology, seasonality or severity of severe acute respiratory infections (SARI) in the Eastern Mediterranean Region.

**Methods:**

Sentinel surveillance for SARI was conducted from December 2007 through February 2014 at 20 hospitals in Egypt, Jordan, Oman, Qatar and Yemen. Nasopharyngeal and oropharyngeal swabs were collected from hospitalized patients meeting SARI case definitions and were analyzed for infection with influenza, respiratory syncytial virus (RSV), adenovirus (AdV), human metapneumovirus (hMPV) and human parainfluenza virus types 1–3 (hPIV1-3). We analyzed surveillance data to calculate positivity rates for viral respiratory pathogens, describe the seasonality of those pathogens and determine which pathogens were responsible for more severe outcomes requiring ventilation and/or intensive care and/or resulting in death.

**Results:**

At least one viral respiratory pathogen was detected in 8,753/28,508 (30.7%) samples tested for at least one pathogen and 3,497/9,315 (37.5%) of samples tested for all pathogens–influenza in 3,345/28,438 (11.8%), RSV in 3,942/24,503 (16.1%), AdV in 923/9,402 (9.8%), hMPV in 617/9,384 (6.6%), hPIV1 in 159/9,402 (1.7%), hPIV2 in 85/9,402 (0.9%) and hPIV3 in 365/9,402 (3.9%). Multiple pathogens were identified in 501/9,316 (5.4%) participants tested for all pathogens. Monthly variation, indicating seasonal differences in levels of infection, was observed for all pathogens. Participants with hMPV infections and participants less than five years of age were significantly less likely than participants not infected with hMPV and those older than five years of age, respectively, to experience a severe outcome, while participants with a pre-existing chronic disease were at increased risk of a severe outcome, compared to those with no reported pre-existing chronic disease.

**Conclusions:**

Viral respiratory pathogens are common among SARI patients in the Eastern Mediterranean Region. Ongoing surveillance is important to monitor changes in the etiology, seasonality and severity of pathogens of interest.

## Introduction

Acute respiratory infections (ARI) are the most common cause of morbidity worldwide [1, 2] and the leading infectious cause of death for children under five years of age [3]. In 2010, ARI were responsible for nearly 1.4 million deaths in children under five years of age, 18.3% of total deaths in this age group [3].

Viral and bacterial etiologies of ARI cannot be distinguished clinically [4], so the microbiological etiology of ARI is often unknown [5]. Data suggest the majority of ARI have a viral etiology [6, 7], yet little attention was given to viral respiratory pathogens other than influenza for many years [5] so the relative impact of these etiologies is poorly understood [6]. Yet the role of these viruses in ARI morbidity and mortality is becoming increasingly apparent as diagnostics improve and become more widely available.

Surveillance for severe acute respiratory infections (SARI) offers an opportunity to examine infections at the more serious end of the spectrum of ARI by focusing on hospitalized patients. Investigating the etiology, seasonality and severity of these infections is key to understanding disease that requires hospital admission and therefore is responsible for considerable morbidity and mortality, as well as a substantial burden on both individual patients and the broader healthcare system.

The epidemiology and impact of many viral respiratory pathogens have been described in temperate climates [7–11], but published data from the Eastern Mediterranean Region are limited. In 2007, the Eastern Mediterranean Acute Respiratory Infection Surveillance (EMARIS) network was established and initiated sentinel-site surveillance for SARI through collaboration by Ministries of Health in Egypt, Jordan and Oman in partnership with the U.S. Centers for Disease Control and Prevention (CDC), U.S. Naval Medical Research Unit No. 3 (NAMRU-3), and the World Health Organization (WHO). Over the next five years, surveillance efforts expanded to include collaborations with Ministries of Health in Qatar and Yemen.

We analyzed surveillance data from SARI patients enrolled at participating hospitals from December 2007 through February 2014 to calculate proportions of positive samples for viral respiratory pathogens, describe the seasonality of those pathogens, and determine which pathogens were responsible for more severe outcomes requiring ventilation or intensive care or resulting in death. Specific pathogens of interest included influenza, respiratory syncytial virus (RSV), adenovirus (AdV), human metapneumovirus (hMPV) and human parainfluenza virus types 1–3 (hPIV1-3), as covered by the core respiratory diagnostic panel of CDC’s International Emerging Infections Program (IEIP) [12].

## Materials and methods

### Surveillance sites

The current analysis includes data from twenty hospitals conducting SARI surveillance in Egypt, Jordan, Oman, Qatar and Yemen. Sentinel surveillance for SARI was established in December 2007 at eight infectious disease hospitals in Egypt. One month later, surveillance began at four general hospitals in Jordan and three general hospitals in Oman. In January 2010, surveillance activities in Egypt expanded to include Cairo University hospitals and Ain Shams University hospitals. In August 2010, Qatar began SARI surveillance in the country’s main hospital, and Yemen began SARI surveillance at two general hospitals in October 2010.

### Participant enrollment

All sentinel hospitals adopted a standardized methodology for SARI surveillance. Hospital surveillance teams screened all hospitalized patients with respiratory disease to assess whether patients met a syndromic case definition for SARI. Due to an annual review by investigators and updates to WHO guidelines, the case definition for SARI evolved over the study period (S1 Table). From 2007 through 2009, the WHO SARI case definition (2006) was used [13]. During 2010–2011, the WHO SARI case definition was expanded to include cases of any age greater than 31 days, cases that met the CDC IEIP pneumonia case definition [14] and any case that a clinician suspected of SARI. In January 2012 the revised WHO SARI case definition (2011) [15] was adopted, and cases with clinical suspicion of SARI continued to be enrolled [16].

Eligible patients who met the SARI case definition were enrolled in the study, and either written or oral informed consent (depending on country of enrollment) was obtained from patients and/or their parents/guardians. A standardized questionnaire was used to collect data on patient demographics, medical history, clinical signs and symptoms, treatment, clinical course and outcome. Nasopharyngeal (NP) and oropharyngeal (OP) swabs were collected and combined in a single vial of viral transport media (VTM) from consenting patients.

### Laboratory procedures

Samples were tested by real time reverse transcriptase polymerase chain reaction (rtRT-PCR) to detect viral ribonucleic acid (RNA) for influenza virus (A or B) at the national central public health laboratories, and aliquots of respiratory samples were then shipped to NAMRU-3 in Cairo, Egypt. At NAMRU-3, total nucleic acid (TNA) was extracted from 200μl of each sample using MagMAX™ Pathogen RNA/DNA Kit with the MagMAX™ Express-96 Deep Well Magnetic Particle Processor (Applied Biosystems®). For each sample, the human RNase P gene was tested as an internal positive control to ensure proper sample collection and nucleic acid extraction. TNA analyzed by polymerase chain reaction (PCR) to identify viral deoxyribonucleic acid (DNA) for adenovirus (AdV) and rtRT-PCR to detect viral RNA for respiratory syncytial virus (RSV), human metapneumnovirus (hMPV) and human parainfluenza virus types 1–3 (hPIV1-3) [17]. Primers and probes used were provided by the Gastroenteritis and Respiratory Viruses Laboratory Branch of the National Center for Immunization and Respiratory Diseases at the CDC.

Questionnaire data were entered into a Microsoft Access database by data managers based either at the hospital or at the central Ministry of Health. Laboratory data were recorded in a Microsoft Excel spreadsheet by laboratory technicians. Questionnaire and laboratory data were linked using a unique study identification number assigned to each participant at enrollment.

### Data analysis

Data were analyzed using SAS software, version 9.3, of the SAS System for Windows (SAS Institute Inc., Cary, North Carolina). Proportions of positive samples were calculated with Clopper-Pearson confidence intervals [18]. Seasonality was examined by plotting the proportion of positive samples by monthly intervals for each pathogen of interest. The chi-squared test for heterogeneity was then used to assess the statistical significance of monthly variance in the proportion of positive samples. Indicators of severe disease were assessed for each pathogen of interest using Mantel-Haenszel estimates to calculate odds ratios and confidence intervals and the Mantel-Haenszel chi-squared test to assess statistical significance [19]. Logistic regression was used to examine associations between viral respiratory pathogens and severe outcomes, defined as illness requiring ventilation or intensive care or resulting in death, while controlling for demographic and clinical characteristics. Only variables with statistically significant univariate association with severe outcomes were included in multivariate regression analysis.

### Ethics approval

Prior to study initiation, the study protocol was reviewed and approved by Institutional Review Boards (IRBs) at CDC and NAMRU-3, as well as by host country Ministries of Health, in compliance with all applicable federal U.S. regulations governing the protection of human subjects and regulations of other participating institutions. IRBs and host country regulators granted a waiver of documentation of informed consent and allowed a process of verbal informed consent to be used due to the minimal risk faced by patients’ participation in the study. The verbal consent process involved surveillance teams explaining the study to patients, providing written study information to patients, allowing patients to consider participation and ask questions. Consent (or lack thereof) for study participation and future use of specimens was documented for all participants on the study consent form and the study questionnaire.

## Results

### Etiology

Of 32,022 participants (14,291 < 5 years, 16,208 ≥ 5 years, 1,523 age unknown) enrolled in SARI surveillance between December 2007 and February 2014, 28,508 (89.0%, 12,612 < 5 years, 14,472 ≥ 5 years, 1,424 age unknown) had an appropriate sample collected and tested for at least one viral respiratory pathogen (influenza, RSV, AdV, hMPV, hPIV1, hPIV2 and/or hPIV3). There were 9,315 (29.1%, 5,181 < 5 years, 3,846 ≥ 5 years, 288 age unknown) participants with samples for which testing for the full panel of viral respiratory pathogens was performed.

Viral nucleic acid for at least one viral respiratory pathogen was detected in 8,753 (30.7%, 95% CI 30.2–31.2%, 5,046 < 5 years, 3,309 ≥ 5 years, 398 age unknown) samples tested for at least one pathogen and 3,497 (37.5%, 95% CI 36.6–38.5%, 2,822 < 5 years, 614 ≥ 5 years, 61 age unknown) samples tested for the full panel of pathogens. Influenza was identified in 3,345/28,438 (11.8%, 95% CI 11.4–12.1%) participants, RSV in 3,942/24,503 (16.1%, 95% CI 15.6–16.6%), AdV in 923/9,402 (9.8%, 95% CI 9.2–10.4%), hMPV in 617/9,384 (6.6%, 95% CI 6.1–7.1%), hPIV1 in 159/9,402 (1.7%, 95% CI 1.4–2.0%), hPIV2 in 85/9,402 (0.9%, 95% CI 0.7–1.1%) and hPIV3 in 365/9,402 (3.9%, 95% CI 3.5–4.3%) (Table 1).

**Table 1:** Proportion of tested SARI cases positive for viral respiratory pathogens (influenza, RSV, AdV, hMPV, hPIV1, hPIV2, hPIV3) by country, year, age and sex.

	SARI cases			Influenza			RSV			AdV			hMPV			hPIV1			hPIV2			hPIV3			Any coinfection		
	n	%	(95% CI)	n	%	(95% CI)	n	%	(95% CI)	n	%	(95% CI)	n	%	(95% CI)	n	%	(95% CI)	n	%	(95% CI)	n	%	(95% CI)	n	%	(95% CI)
Country																											
Egypt	20753/32022	64.8	(64.3–65.3)	2766/18454	15.0	(14.5–15.5)	1973/18247	10.8	(10.4–11.3)	526/6516	8.1	(7.4–8.8)	446/6520	6.8	(6.2–7.5)	107/6516	1.6	(1.3–2.0)	51/6516	0.8	(0.6–1.0)	252/6516	3.9	(3.4–4.4)	266/6506	4.1	(3.6–4.6)
Jordan	3366/32022	10.5	(10.2–10.9)	332/3063	10.8	(9.8–12.0)	1015/3051	33.3	(31.6–35.0)	80/855	9.4	(7.5–11.5)	68/855	8.0	(6.2–10.0)	13/855	1.5	(0.8–2.6)	16/855	1.9	(1.1–3.0)	37/855	4.3	(3.1–5.9)	55/855	6.4	(4.9–8.3)
Oman	6084/32022	19.0	(18.6–19.4)	154/5505	2.8	(2.4–3.3)	579/1839	31.5	(29.4–33.7)	114/634	18.0	(15.1–21.2)	68/634	10.7	(8.4–13.4)	12/634	1.9	(1.0–3.3)	7/634	1.1	(0.4–2.3)	29/634	4.6	(3.1–6.5)	64/634	10.1	(7.9–12.7)
Qatar	518/32022	1.6	(1.5–1.8)	12/333	3.6	(1.9–6.2)	23/325	7.1	(4.5–10.4)	12/356	3.4	(1.8–5.8)	15/356	4.2	(2.4–6.9)	3/356	0.8	(0.2–2.4)	3/356	0.8	(0.2–2.4)	3/356	0.8	(0.2–2.4)	2/308	0.6	(0.1–2.3)
Yemen	1301/32022	4.1	(3.8–4.3)	81/1083	7.5	(6.0–9.2)	352/1041	33.8	(30.9–36.8)	191/1041	18.3	(16.0–20.8)	20/1019	2.0	(1.2–3.0)	24/1041	2.3	(1.5–3.4)	8/1041	0.8	(0.3–1.5)	44/1041	4.2	(3.1–5.6)	114/1012	11.3	(9.4–13.4)
				χ^2^ = 654.37, df = 4, p<0.01			χ^2^ = 1627.96, df = 4, p<0.01			χ^2^ = 172.61, df = 4, p<0.01			χ^2^ = 59.69, df = 4, p<0.01			χ^2^ = 4.30, df = 4, p = 0.37			χ^2^ = 10.51, df = 4, p = 0.03			χ^2^ = 10.41, df = 4, p = 0.03			χ^2^ = 133.29, df = 4, p<0.01		
Year																											
2007–2008	1816/32022	5.7	(5.4–5.9)	106/1677	6.3	(5.3–7.6)	263/1667	15.8	(14.1–17.6)	150/1677	8.9	(7.6–10.4)	108/1677	6.4	(5.3–7.7)	3/1677	0.2	(0.0–0.5)	17/1677	1.0	(0.6–1.6)	81/1677	4.8	(3.9–6.0)	49/1667	2.9	(2.2–3.9)
2008–2009	2225/32022	6.9	(6.7–7.2)	172/2114	8.1	(7.0–9.4)	406/2114	19.2	(17.6–20.9)	232/2114	11.0	(9.7–12.4)	146/2114	6.9	(5.9–8.1)	70/2114	3.3	(2.6–4.2)	30/2114	1.4	(1.0–2.0)	84/2114	4.0	(3.2–4.9)	121/2114	5.7	(4.8–6.8)
2009–2010	5630/32022	17.6	(17.2–18.0)	425/5059	8.4	(7.7–9.2)	829/4791	17.3	(16.3–18.4)	146/1916	7.6	(6.5–8.9)	239/1917	12.5	(11.0–14.0)	15/1916	0.8	(0.4–1.3)	1/1920	0.1	(0.0–0.3)	54/1916	2.8	(2.1–3.7)	103/1916	5.4	(4.4–6.5)
2010–2011	8698/32022	27.2	(26.7–27.7)	1110/8045	13.8	(13.1–14.6)	1070/6726	15.9	(15.1–16.8)	131/1529	8.6	(7.2–10.1)	67/1528	4.4	(3.4–5.5)	29/1529	1.9	(1.3–2.7)	13/1549	0.8	(0.4–1.4)	70/1529	4.6	(3.6–5.8)	69/1524	4.5	(3.5–5.7)
2011–2012	5053/32022	15.8	(15.4–16.2)	506/4681	10.8	(10.0–11.7)	501/3803	13.2	(12.1–14.3)	114/1215	9.4	(7.8–11.2)	43/1218	3.5	(2.6–4.7)	21/1215	1.7	(1.1–2.6)	17/1222	1.4	(0.8–2.2)	35/1215	2.9	(2.0–4.0)	73/1212	6.0	(4.8–7.5)
2012–2013	5647/32022	17.6	(17.2–18.1)	483/4405	11.0	(10.1–11.9)	537/3271	16.4	(15.2–17.7)	102/668	15.3	(12.6–18.2)	10/639	1.6	(0.7–2.9)	14/668	2.1	(1.2–3.5)	4/651	0.6	(0.2–1.6)	29/668	4.3	(2.9–6.2)	58/615	9.4	(7.2–12.0)
2013–2014	2639/32022	8.2	(7.9–8.5)	476/2201	21.6	(20.0–23.4)	271/1884	14.4	(12.9–16.0)	48/259	18.5	(14.0–23.8)	4/269	1.5	(0.4–3.8)	7/259	2.7	(1.1–5.5)	3/259	1.2	(0.2–3.4)	12/259	4.6	(2.4–8.0)	28/259	10.8	(7.3–15.2)
Unknown	314/32022	1.0	(0.9–1.1)	67/256	26.2	(21.2–31.9)	65/247	26.3	(20.9–32.3)	0/24	0.0	(0.0–14.3)	0/22	0.0	(0.0–15.4)	0/24	0.0	(0.0–14.3)	0/10	0.0	(0.0–30.9)	0/24	0.0	(0.0–14.3)	0/8	0.0	(0.0–36.9)
				χ^2^ = 378.13, df = 6, p<0.01			χ^2^ = 49.04, df = 6, p<0.01			χ^2^ = 62.55, df = 6, p<0.01			χ^2^ = 176.15, df = 6, p<0.01			χ^2^ = 68.43, df = 6, p<0.01			χ^2^ = 26.12, df = 6, p<0.01			χ^2^ = 15.87, df = 6, p = 0.01			χ^2^ = 57.96, df = 6, p<0.01		
Age																											
<5 years	14291/32022	44.6	(44.1–45.2)	762/12607	6.0	(5.6–6.5)	3110/10018	31.0	(20.1–32.0)	762/5210	14.6	(13.7–15.6)	425/5193	8.2	(7.4–9.0)	129/5210	2.5	(2.1–2.9)	67/5212	1.3	(1.0–1.6)	303/5210	5.8	(5.2–6.5)	422/5181	8.1	(7.4–8.9)
≥ 5 years	16208/32022	50.6	(50.1–51.2)	2377/14439	16.5	(15.9–17.1)	655/13134	5.0	(4.6–5.4)	145/3868	3.7	(3.2–4.4)	185/3869	4.8	(4.1–5.5)	22/3868	0.6	(0.3–0.9)	16/3895	0.4	(0.2–0.7)	56/3868	1.4	(1.1–1.9)	75/3846	2.0	(1.5–2.4)
Unknown	1523/32022	4.8	(4.5–5.0)	206/1392	14.8	(13.0–16.8)	177/1351	13.1	(11.4–15.2)	16/324	4.9	(2.9–7.9)	7/322	2.2	(0.9–4.4)	8/324	2.5	(1.1–4.8)	2/295	0.7	(0.1–2.4)	6/324	1.9	(0.7–4.0)	4/288	1.4	(0.3–3.5)
				χ^2^ = 712.05, df = 1, p<0.01			χ^2^ = 2833.62, df = 1, p<0.01			χ^2^ = 292.05, df = 1, p<0.01			χ^2^ = 40.88, df = 1, p<0.01			χ^2^ = 49.37, df = 1, p<0.01			χ^2^ = 18.89, df = 1, p<0.01			χ^2^ = 111.51, df = 1, p<0.01			χ^2^ = 162.84, df = 1, p<0.01		
Sex																											
Male	16797/32022	52.5	(51.9–53.0)	1684/14803	11.4	(10.9–11.9)	2092/12911	16.2	(15.6–16.9)	525/5268	10.0	(9.2–10.8)	327/5260	6.2	(5.6–6.9)	94/5268	1.8	(1.4–2.2)	50/5270	0.9	(0.7–1.2)	208/5268	3.9	(3.4–4.5)	212/3809	5.6	(4.9–6.3)
Female	13883/32022	43.4	(42.8–43.9)	1487/12378	12.0	(11.5–12.6)	1686/10373	16.3	(15.5–17.0)	382/3844	9.9	(9.0–10.9)	281/3834	7.3	(6.5–8.2)	57/3844	1.5	(1.1–1.9)	34/3838	0.9	(0.6–1.2)	150/3844	3.9	(3.3–4.6)	285/5218	5.5	(4.9–6.1)
Unknown	1342/32022	4.2	(4.0–4.4)	174/1257	13.8	(12.0–15.9)	164/1219	13.5	(11.6–15.5)	16/290	5.5	(3.2–8.8)	9/290	3.1	(1.4–5.8)	8/290	2.8	(1.2–5.4)	1/294	0.3	(0.0–1.9)	7/290	2.4	(1.0–4.9)	4/288	1.4	(0.4–3.5)
				χ^2^ = 2.66, df = 1, p = 0.10			χ^2^ = 0.01, df = 1, p = 0.92			χ^2^<0.01, df = 1, p = 0.96			χ^2^ = 4.40, df = 1, p = 0.04			χ^2^ = 1.24, df = 1, p = 0.27			χ^2^ = 0.10, df = 1, p = 0.76			χ^2^ = 0.01, df = 1, p = 0.91			χ^2^ = 0.05, df = 1, p = 0.83		
TOTAL				3345/28438	11.8	(11.4–12.1)	3,942 /24503	16.1	(15.6–16.6)	923/9402	9.8	(9.2–10.4)	617/9384	6.6	(6.1–7.1)	159/9402	1.7	(1.4–2.0)	85/9402	0.9	(0.7–1.1)	365/9402	3.9	(3.5–4.3)	501/9315	5.4	(4.9–5.9)

RSV: respiratory syncytial virus, AdV: adenovirus, hMPV: human metapneumovirus, hPIV1-3: human parainfluenza virus types 1–3, CI: confidence interval, df: degrees of freedom, p: p-value

RSV was the most common pathogen detected in each country except Egypt, where influenza was the most common pathogen detected. The proportion of positive samples varied across countries for all pathogens (p-values range from <0.01 to 0.03) except hPIV1 (p = 0.37) and across years for all pathogens (p-values range from <0.01 to 0.01). hMPV was the only pathogen for which different proportions of positive samples were found by sex (p = 0.04). All pathogens were more common in children under the age of five years than in older participants (all p <0.01), except influenza, which was significantly more common in participants at least five years of age (16.5% vs. 6.0%, p<0.01).

Multiple viral respiratory infections were identified in 501 (5.4%) samples tested for all pathogens– 477 (5.1%) with two pathogens, 21 (0.2%) with three pathogens and three (<0.1%) with four pathogens (Table 2). AdV was the most common virus present in co-infections (n = 362), followed by RSV (n = 329), hMPV (n = 138), influenza (n = 121), hPIV3 (n = 97), hPIV1 (n = 45) and hPIV2 (n = 24). The proportion of participants with co-infections varied across countries and years and was significantly higher among children under the age of five years than among older participants (8.1% vs. 2.0%, p<0.01) (Table 1).

**Table 2:** Coinfections by viral respiratory pathogen (n = 501). Includes 5 patients with influenza, RSV and AdV; 3 with influenza, RSV and hMPV; 3 with RSV, AdV and hMPV; 3 with RSV, AdV and hPIV1; 2 with RSV, AdV and hPIV3; 2 with AdV, hMPV and hPIV3; 1 with influenza, AdV and hMPV; 1 with RSV, AdV and hPIV2; 1 with RSV, hPIV1 and hPIV2; 1 with influenza, RSV, AdV and hMPV; 1 with RSV, AdV, hMPV and hPIV3; 1 with RSV, AdV, hPIV1 and hPIV3.

	Influenza			RSV			AdV			hMPV			hPIV1			hPIV2			hPIV3		
	n	%	(95% CI)	n	%	(95% CI)	n	%	(95% CI)	n	%	(95% CI)	n	%	(95% CI)	n	%	(95% CI)	n	%	(95% CI)
Influenza	-			64	0.7	(0.5–0.9)	30	0.3	(0.2–0.5)	18	0.2	(0.1–0.3)	1	0	(0.0–0.1)	2	0	(0.0–0.1)	6	0.1	(0.0–0.1)
RSV	64	0.7	(0.5–0.9)	-			181	1.9	(1.7–2.2)	37	0.4	(0.3–0.5)	13	0.1	(0.1–0.2)	12	0.1	(0.1–0.2)	22	0.2	(0.1–0.4)
AdV	30	0.3	(0.2–0.5)	181	1.9	(1.7–2.2)	-			66	0.7	(0.5–0.9)	24	0.3	(0.2–0.4)	7	0.1	(0.0–0.2)	54	0.6	(0.4–0.8)
hMPV	18	0.2	(0.1–0.3)	37	0.4	(0.3–0.5)	66	0.7	(0.5–0.9)	-			5	0.1	(0.0–0.1)	0	0	(0.0–0.0)	12	0.1	(0.1–0.2)
hPIV1	1	0	(0.0–0.1)	13	0.1	(0.1–0.2)	24	0.3	(0.2–0.4)	5	0.1	(0.0–0.1)	-			1	0	(0.0–0.1)	1	0	(0.0–0.1)
hPIV2	2	0	(0.0–0.1)	12	0.1	(0.1–0.2)	7	0.1	(0.0–0.2)	0	0	(0.0–0.0)	1	0	(0.0–0.1)	-			2	0	(0.0–0.1)
hPIV3	6	0.1	(0.0–0.1)	22	0.2	(0.1–0.4)	54	0.6	(0.4–0.8)	12	0.1	(0.1–0.2)	1	0	(0.0–0.1)	2	0	(0.0–0.1)	-		

RSV: respiratory syncytial virus, AdV: adenovirus, hMPV: human metapneumovirus, hPIV1-3: human parainfluenza virus types 1–3, CI: confidence interval

### Seasonality

**Fig 1:**
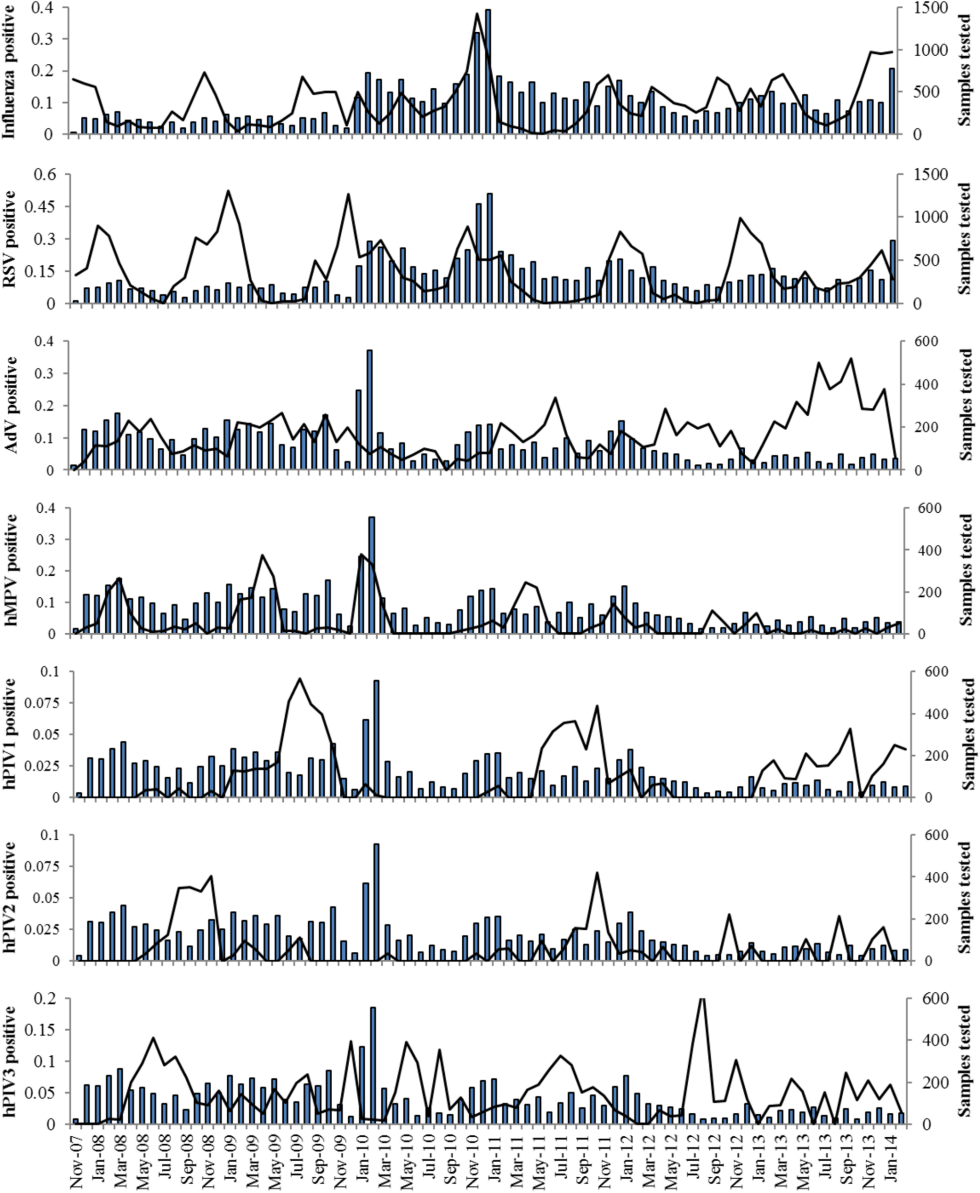
Seasonality of viral respiratory pathogens Bars indicates the total number of samples tested for each pathogen (shown on right vertical axis). Lines indicate the proportion of positive samples for each pathogen (shown on left vertical axis). Vertical axes vary between pathogens. Horizontal axes indicate time in monthly intervals from November 2007 through February 2014.

Patterns of seasonality in the number of SARI cases tested for at least one viral respiratory pathogen and the proportion of positive samples for each pathogen are shown in Fig 1.

**Fig 2:**
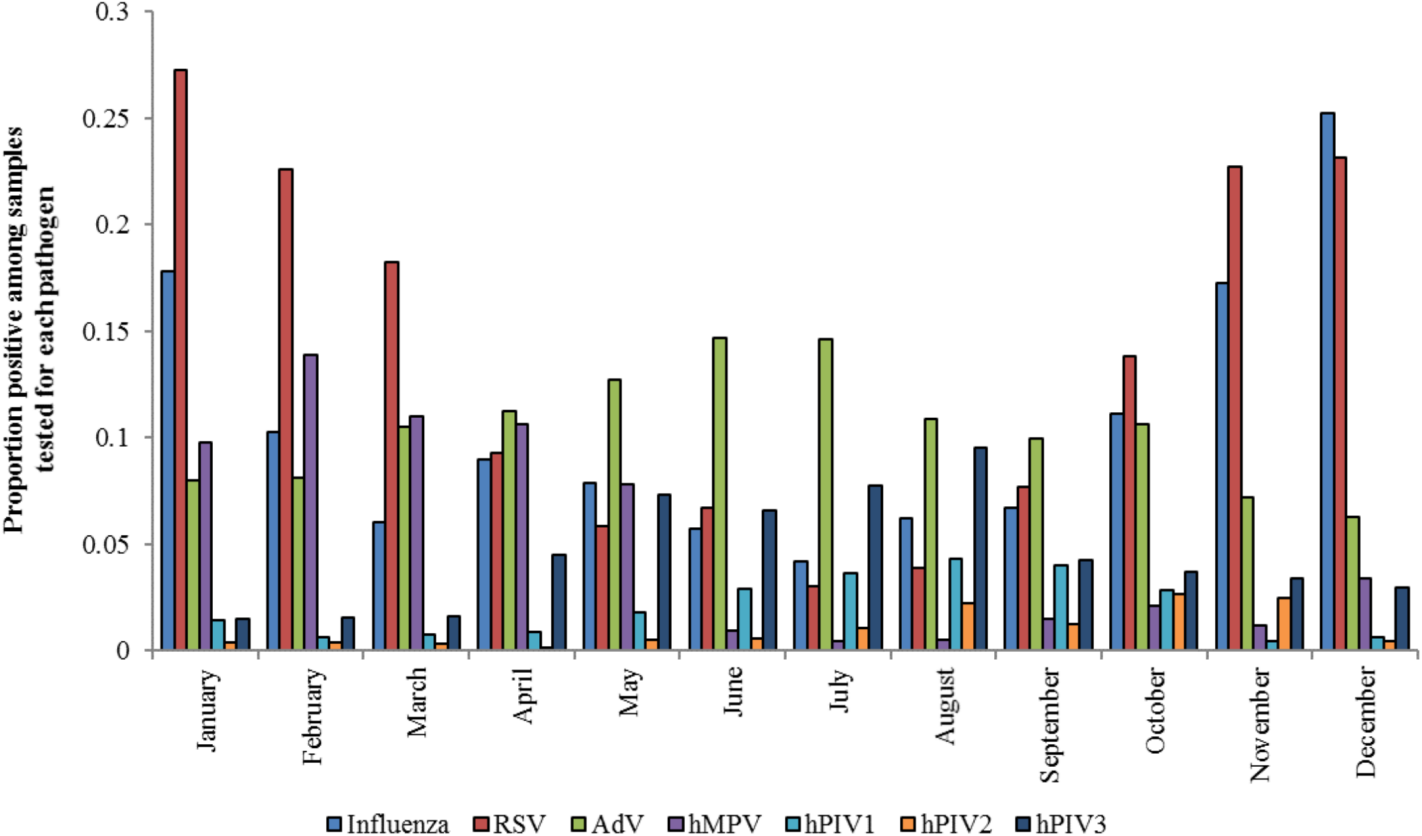
Proportion of positive samples for each viral respiratory pathogen by month. Proportion of positive samples out of total samples tested for each pathogen are shown.

The proportion of positive samples identified during each month is shown in Fig 2. The proportion of samples positive for each pathogen of interest varied significantly by month (influenza χ^2^ = 1098, p<0.01; RSV χ^2^ = 1263, p<0.01; AdV χ^2^ = 52, p<0.01; hMPV χ^2^ = 365, p<0.01; hPIV1 χ^2^ = 90, p<0.01; hPIV2 χ^2^ = 76, p<0.01; hPIV3 χ^2^ = 158, p<0.01; all with 11 degrees of freedom).

### Severity

No infections were independently associated with increased severity of illness, as indicated by illness requiring ventilation and/or intensive care and/or resulting in death (Table 3). There was strong evidence that participants with influenza, RSV, AdV or hMPV infection were less likely to experience a severe outcome than participants not infected with each of these pathogens (influenza OR 0.70, 95% CI 0.60–0.81, p<0.01; RSV OR 0.82, 95% CI 0.71–0.94, p<0.01; AdV OR 0.71, 95% CI 0.55–0.91, p<0.01; hMPV OR 0.27, 95% CI 0.18–0.41, p<0.01). Participants with multiple infections were no more likely than participants with no infection or infection with a single pathogen to experience severe outcomes (OR 1.23, 95% CI 0.92–1.64, p = 0.16).

**Table 3:** Indicators of severity of disease by pathogen (influenza, RSV, AdV, hMPV, PIV1, PIV2, PIV3 and any coinfection). Severe outcome is defined as illness requiring ventilation or intensive care or resulting in death. For each pathogen of interest, tested SARI patients with a positive result for that pathogen were compared to a reference group of tested SARI patients with a negative result for that pathogen. For “any coinfection”, tested SARI patients with any coinfection were compared to a reference group of tested SARI patients with test results for all pathogens of interest and no identified coinfection.

	SARI cases		Influenza				RSV				AdV				hMPV				hPIV1				hPIV2				hPIV3				Any coinfection			
	n	%	n	%	Odds ratio	p-value	n	%	Odds ratio	p-value	n	%	Odds ratio	p-value	n	%	Odds ratio	p-value	n	%	Odds ratio	p-value	n	%	Odds ratio	p-value	n	%	Odds ratio	p-value	n	%	Odds ratio	p-value
All participants																																		
Ventilation	900/25210	3.6%	75/2832	2.6%	0.76 (0.59–0.96)	0.02	74/2844	2.6%	0.70 (0.54–0.89)	<0.01	27/455	5.9%	0.61 (0.41–0.91)	0.02	11/343	3.2%	0.31 (0.17–0.58)	<0.01	2/64	3.1%	0.32 (0.08–1.32)	0.10	4/36	11.1%	1.26 (0.44–3.58)	0.67	17/178	9.6%	1.06 (0.64–1.77)	0.81	20/434	4.6%	1.31 (0.83–2.07)	0.24
Intensive care	2282/25211	9.1%	182/2835	6.4%	0.69 (0.59–0.81)	<0.01	224/2839	7.9%	0.89 (0.77–1.03)	0.12	75/449	16.7%	0.75 (0.58–0.97)	0.03	19/343	5.5%	0.21 (0.13–0.34)	<0.01	9/63	14.3%	0.64 (0.31–1.29)	0.21	6/36	16.7%	0.77 (0.32–1.85)	0.55	32/175	18.3%	0.85 (0.58–1.26)	0.42	49/431	11.4%	1.30 (0.96–1.75)	0.09
Death	971/27433	3.5%	81/2828	2.9%	0.83 (0.66–1.05)	0.13	71/3457	2.1%	0.55 (0.43–0.70)	<0.01	31/798	3.9%	0.69 (0.48–1.01)	0.05	13/486	2.7%	0.47 (0.27–0.82)	<0.01	2/133	1.5%	0.27 (0.07–1.08)	0.05	2/71	2.8%	0.51 (0.13–2.10)	0.34	16/312	5.1%	0.96 (0.57–1.60)	0.86	19/560	3.4%	0.96 (0.60–1.52)	0.85
Severe outcome	2673/23467	11.4%	209/2551	8.2%	0.70 (0.60–0.81)	<0.01	254/2713	9.4%	0.82 (0.71–0.94)	<0.01	81/426	19.0%	0.71 (0.55–0.91)	<0.01	25/293	8.5%	0.27 (0.18–0.41)	<0.01	10/61	16.4%	0.60 (0.31–1.19)	0.14	6/34	17.6%	0.66 (0.27–1.61)	0.36	36/174	20.7%	0.80 (0.55–1.16)	0.25	54/397	13.6%	1.23 (0.92–1.64)	0.16
Participants aged < 5 years																																		
Ventilation	297/11249	2.6%	16/636	2.5%	1.06 (0.63–1.77)	0.82	48/2243	2.1%	0.72 (0.52–1.01)	0.05	23/378	6.1%	1.03 (0.65–1.63)	0.90	7/219	3.2%	0.50 (0.23–1.08)	0.07	2/56	3.6%	0.58 (0.14–2.41)	0.45	4/28	14.3%	2.68 (0.92–7.83)	0.06	13/150	8.7%	1.55 (0.86–2.80)	0.15	15/314	4.8%	1.90 (1.11–3.23)	0.02
Intensive care	900/11235	8.0%	35/363	9.6%	0.69 (0.49–0.98)	0.04	162/2237	7.2%	1.01 (0.83–1.22)	0.94	62/373	16.6%	1.35 (1.00–1.82)	0.05	8/219	3.7%	0.23 (0.11–0.47)	<0.01	8/56	14.3%	1.08 (0.51–2.30)	0.85	5/28	17.9%	1.41 (0.53–3.74)	0.49	22/148	14.9%	1.14 (0.71–1.81)	0.59	37/312	11.9%	1.57 (1.11–2.23)	0.01
Death	334/13110	2.5%	19/701	2.7%	1.18 (0.73–1.89)	0.50	51/2825	1.8%	0.66 (0.48–0.91)	0.01	28/675	4.1%	1.15 (0.76–1.74)	0.51	9/343	2.6%	0.68 (0.34–1.34)	0.26	2/115	1.7%	0.45 (0.11–1.85)	0.26	2/59	3.4%	0.91 (0.22–3.76)	0.90	10/268	3.7%	1.01 (0.53–1.93)	0.98	16/433	3.7%	1.49 (0.89–2.49)	0.12
Severe outcome	1039/10768	9.6%	44/594	7.4%	0.77 (0.56–1.06)	0.10	187/2151	8.7%	0.97 (0.81–1.16)	0.71	68/357	19.0%	1.29 (0.96–1.71)	0.09	12/192	6.3%	0.33 (0.18–0.60)	<0.01	9/55	16.4%	1.03 (0.50–2.12)	0.94	5/27	18.5%	1.20 (0.45–3.18)	0.72	26/147	17.7%	1.14 (0.74–1.77)	0.56	42/291	14.4%	1.60 (1.15–2.24)	0.01
Participants aged ≥ 5 years																																		
Ventilation	563/13619	4.1%	57/2160	2.6%	0.62 (0.47–0.82)	<0.01	23/561	4.1%	1.08 (0.70–1.65)	0.73	4/73	5.5%	0.41 (0.15–1.15)	0.08	4/124	3.2%	0.23 (0.08–0.63)	<0.01	0/8	0.0%	<0.01 (<0.01–>999.9)	0.29	0/7	0.0%	<0.01 (<0.01–>999.9)	0.33	4/28	14.3%	1.22 (0.42–3.55)	0.71	5/113	4.4%	1.07 (0.44–2.64)	0.88
Intensive care	1323/13631	9.7%	142/2163	6.6%	0.65 (0.54–0.77)	<0.01	59/561	10.5%	1.16 (0.88–1.53)	0.30	12/72	16.7%	0.47 (0.25–0.88)	0.02	11/124	8.9%	0.22 (0.12–0.41)	<0.01	1/7	14.3%	0.40 (0.05–3.32)	0.38	1/7	14.3%	0.41 (0.05–3.37)	0.39	10/27	37.0%	1.42 (0.65–3.12)	0.38	12/112	10.7%	1.12 (0.61–2.04)	0.72
Death	598/14035	4.3%	61/2096	2.9%	0.68 (0.52–0.89)	<0.01	18/595	3.0%	0.76 (0.47–1.22)	0.25	3/119	2.5%	0.35 (0.11–1.10)	0.06	4/143	2.8%	0.39 (0.14–1.06)	0.05	0/18	0.0%	<0.01 (<0.01–>999.9)	0.25	0/12	0.0%	<0.01 (<0.01–>999.9)	0.35	6/44	13.6%	2.20 (0.92–5.27)	0.07	3/120	2.5%	0.57 (0.18–1.81)	0.34
Severe outcome	1565/12432	12.6%	160/1926	8.3%	0.62 (0.52–0.74)	<0.01	63/526	12.0%	1.00 (0.77–1.32)	0.98	12/65	18.5%	0.42 (0.22–0.79)	<0.01	13/101	12.9%	0.27 (0.15–0.48)	<0.01	1/6	16.7%	0.38 (0.04–3.25)	0.36	1/7	14.3%	0.32 (0.04–2.65)	0.26	10/27	37.0%	1.12 (0.51–2.46)	0.78	12/99	12.1%	0.96 (0.52–1.76)	0.89

RSV: respiratory syncytial virus, AdV: adenovirus, hMPV: human metapneumovirus, hPIV1-3: human parainfluenza virus types 1–3, OR: odds ratio, CI: confidence interval

When analyses were stratified by age, participants less than five years of age with an hMPV infection were less likely than participants without an hMPV infection to experience a severe outcome (OR 0.33, 95% CI 0.18–0.60, p<0.01), but participants with a coinfection were at greater risk of a severe outcome than participants with no infection or infection with a single pathogen (OR 1.60, 95% CI 1.15–2.24, p = 0.01). Among participants aged five years and older, those with an influenza, AdV or hMPV were less likely to experience a severe outcome than those not infected with each of these viruses (influenza OR 0.62, 95% CI 0.52–0.74, p<0.01; AdV OR 0.42, 95% CI 0.22–0.79, p<0.01; hMPV OR 0.27, 95% CI 0.15–0.48, p<0.01).

Logistic regression was used to further examine associations with severe outcomes in participants for whom testing for the full panel of viral respiratory viruses was performed and for whom data on additional demographics and clinical risk factors variables are complete. In univariate logistic regression examining associations between severe outcomes and viral respiratory pathogens, demographics and clinical risk factors, participants with hMPV infection and participants less than five years of age were less likely to experience a severe outcome than participants not infected with hMPV (OR 0.30, 95% CI 0.16–0.58, p<0.01) and participants aged five years and older (OR 0.45, 95% CI 0.39–0.53, p<0.01), respectively (Table 4). Participants with any reported pre-existing chronic disease and participants with reported care-seeking delay of at least seven days were at greater risk of severe outcome than participants with no reported pre-existing chronic disease (OR 2.57, 95% CI 2.20–3.01, p<0.01) and participants with reported care-seeking delay less than seven days (OR 1.25, 95% CI 1.06–1.47, p = 0.01).

**Table 4:** Univariate and multivariate logistic regression examining predictors of severe outcomes. Includes only individuals for whom testing for the full panel of viral respiratory viruses was performed and for whom data on demographics and clinical risk factors are complete. Severe outcome is defined as illness requiring ventilation or intensive care or resulting in death.

UNIVARIATE ANALYSIS			
		OR (95% CI)	p-value
Influenza	Positive	1.47 (0.91–2.39)	0.12
	Negative	ref.	
RSV	Positive	0.81 (0.64–1.04)	0.10
	Negative	ref.	
AdV	Positive	0.87 (0.67–1.13)	0.30
	Negative	ref.	
hMPV	Positive	0.30 (0.16–0.58)	<0.01
	Negative	ref.	
hPIV1	Positive	1.04 (0.49–2.23)	0.92
	Negative	ref.	
hPIV2	Positive	1.12 (0.38–3.29)	0.84
	Negative	ref.	
hPIV3	Positive	1.21 (0.77–1.91)	0.41
	Negative	ref.	
Age	< 5 years	0.45 (0.39–0.53)	<0.01
	≥ 5 years	ref.	
Sex	Male	ref.	
	Female	1.11 (0.95–1.29)	0.20
Pre-existing chronic disease	None	ref.	
	Any	2.57 (2.20-3.01)	<0.01
Care-seeking delay	< 7 days	ref.	
	≥ 7 days	1.25 (1.06–1.47)	0.01
MULTIVARIATE ANALYSIS			
		OR (95% CI)	p-value
Influenza	Positive	1.18 (0.86–1.61)	0.30
	Negative	ref.	
RSV	Positive	0.87 (0.70–1.08)	0.21
	Negative	ref.	
AdV	Positive	1.22 (0.92–1.61)	0.17
	Negative	ref.	
hMPV	Positive	0.29 (0.18–0.47)	<0.01
	Negative	ref.	
hPIV1	Positive	0.97 (0.46–2.02)	0.93
	Negative	ref.	
hPIV2	Positive	0.97 (0.34–2.60)	0.94
	Negative	ref.	
hPIV3	Positive	1.04 (0.68–1.59)	0.96
	Negative	ref.	
Age	< 5 years	0.53 (0.44–0.62)	<0.01
	≥ 5 years	ref.	
Pre-existing chronic disease	None	ref.	
	Any	2.34 (1.99–2.76)	<0.01
Care-seeking delay	None	ref.	
	Any	1.05 (0.88–1.25)	0.59

RSV: respiratory syncytial virus, AdV: adenovirus, hMPV: human metapneumovirus, hPIV1-3: human parainfluenza virus types 1–3, OR: odds ratio, CI: confidence interval, ref.: reference group in analysis

Logistic regression was also performed separately for participants less than five years of age and those at least five years of age (Table 5). In univariate analysis among participants less than five years of age, participants with hMPV infection were less likely to experience a severe outcome than participants not infected with hMPV (OR 0.30, 95% CI 0.16–1.86, p<0.01). In this age group, participants with AdV infection, participants less than one year of age, participants with any reported pre-existing chronic disease were more likely to experience a severe outcome than participants not infected with AdV (1.39, 95% CI 1.03–1.86, p = 0.03), participants aged 1–4 years (OR 1.41, 95% CI 1.10–1.80, p<0.01) and participants with no reported pre-existing chronic disease (OR 1.66, 95% CI 1.33–2.07, p<0.01), respectively. These associations remained, and with similar odds ratios, in multivariate regression adjusting for potential confounding.

**Table 5:** Univariate and multivariate logistic regression examining predictors of severe outcomes by age. Includes only individuals for whom testing for the full panel of viral respiratory pathogens was performed and for whom data on predictor variables are complete. Severe outcome is defined as illness requiring ventilation or intensive care or resulting in death. Participants are divided into age less than five years and age five years or older.

UNIVARIATE ANALYSIS					
		AGE < 5 YEARS		AGE ≥ 5 YEARS	
		OR (95% CI)	p-value	OR (95% CI)	p-value
Influenza	Positive	1.47 (0.91–2.39)	0.12	0.92 (0.63–1.35)	0.67
	Negative	ref.		ref.	
RSV	Positive	0.81 (0.64–1.04)	0.10	0.75 (0.49–1.14)	0.17
	Negative	ref.		ref.	
AdV	Positive	1.39 (1.03–1.86)	0.03	0.40 (0.19–0.85)	0.02
	Negative	ref.		ref.	
hMPV	Positive	0.30 (0.16–0.58)	<0.01	0.30 (0.16–0.59)	<0.01
	Negative	ref.		ref.	
hPIV1	Positive	1.04 (0.49–2.23)	0.92	0.63 (0.70–5.64)	0.68
	Negative	ref.		ref.	
hPIV2	Positive	1.12 (0.38–3.29)	0.84	0.63 (0.70–5.64)	0.68
	Negative	ref.		ref.	
hPIV3	Positive	1.21 (0.77–1.91)	0.41	1.08 (0.41–2.83)	0.87
	Negative	ref.		ref.	
Age	< 1 year	1.41 (1.10–1.80)	<0.01	n/a	
	1–4 years	ref.		n/a	
	5–49 years	n/a		ref.	
	≥ 50 years	n/a		2.73 (2.17–3.42)	<0.01
Sex	Male	ref.		ref.	
	Female	1.00 (0.80–1.26)	0.98	1.17 (0.94–1.46)	0.16
Pre-existing chronic disease	None	ref.		ref.	
	Any	1.66 (1.33–2.07)	<0.01	3.43 (2.70–4.37)	<0.01
Care-seeking delay	< 7 days	ref.		ref.	
	≥ 7 days	1.15 (0.90–1.48)	0.26	1.16 (0.92–1.46)	0.22
MULTIVARIATE ANALYSIS					
		AGE < 5 YEARS		AGE ≥ 5 YEARS	
		OR (95% CI)	p-value	OR (95% CI)	p-value
Influenza	Positive	1.63 (0.98–2.67)	0.05	0.97 (0.65–1.46)	0.89
	Negative	ref.		ref.	
RSV	Positive	0.82 (0.63–1.06)	0.12	0.89 (0.57–1.39)	0.59
	Negative	ref.		ref.	
AdV	Positive	1.48 (1.09–2.00)	0.01	0.57 (0.26–1.25)	0.16
	Negative	ref.		ref.	
hMPV	Positive	0.31 (0.16–0.59)	<0.01	0.29 (0.15–0.58)	<0.01
	Negative	ref.		ref.	
hPIV1	Positive	1.04 (0.48–2.25)	0.92	0.93 (0.10–8.74)	0.95
	Negative	ref.		ref.	
hPIV2	Positive	1.14 (0.38–3.41)	0.81	0.66 (0.07–6.37)	0.72
	Negative	ref.		ref.	
hPIV3	Positive	1.04 (0.65–1.66)	0.87	1.33 (0.49–3.66)	0.58
	Negative	ref.		ref.	
Age	< 1 year	1.43 (1.11–1.83)	<0.01	n/a	
	1–4 years	ref.		n/a	
	5–49 years	n/a		ref.	
	≥ 50 years	n/a		2.00 (1.57–2.55)	<0.01
Pre-existing chronic disease	None	ref.		ref.	
	Any	1.69 (1.34–2.12)	<0.01	2.74 (2.12–3.53)	<0.01

RSV: respiratory syncytial virus, AdV: adenovirus, hMPV: human metapneumovirus, hPIV1-3: human parainfluenza virus types 1–3, OR: odds ratio, CI: confidence interval, ref.: reference group in analysis, n/a: not applicable

In univariate analysis among participants aged five and older, AdV and hMPV infection were each associated with lower risk of severe outcome, compared to participants not infected with AdV (OR 0.40, 95% CI 0.19–0.85, p = 0.02) and participants not infected with hMPV (OR 0.30, 95% CI 0.16–0.59, p<0.01), respectively. Within this age group, age greater than 50 years and any reported pre-existing chronic disease were associated with increased risk of severe outcome, compared with participants aged 5–49 years (2.73, 95% CI 2.17–3.42, p<0.01) and participants with no reported pre-existing chronic disease (OR 3.43, 95% CI 2.70–4.37, p<0.01), respectively. In multivariate logistic regression among participants aged five and older, there remained strong evidence that hMPV infection was associated with lower risk of severe outcome, with a similar odds ratio as in univariate analysis. There also remained strong evidence of an association between severe outcome and age greater than 50 years (OR 2.00, 95% CI 1.57–2.55, p<0.01) or reported pre-existing chronic disease (OR 2.74, 95% CI 2.12–3.53, p<0.01), but with lower odds ratios than in univariate analyses, reflecting an association between increased age and reported pre-existing chronic disease.

## Discussion

Results from seven years of SARI surveillance at hospitals in five countries in the Eastern Mediterranean Region provide important insight into the etiology, seasonality and severity of viral respiratory pathogens among hospitalized patients in this region. Influenza, RSV and AdV were the most common pathogens identified among SARI patients tested for viral respiratory pathogens, although levels of infection varied across countries and over years of surveillance. All pathogens examined were more frequently identified in participants less than five years of age than in older participants. Monthly variation, indicating seasonal differences in levels of infection, was observed for all pathogens. Participants with hMPV infections and participants less than five years of age were significantly less likely than participants not infected with hMPV and those older than five years of age, respectively, to experience a severe outcome, while participants with a pre-existing chronic disease were at increased risk of a severe outcome, compared to those with no reported pre-existing chronic disease.

SARI cases under the age of five tested for viral respiratory pathogens were significantly more likely than older patients to be infected with each of the pathogens examined, except influenza. This is not unexpected since these pathogens have a strong association with this age group, while influenza is associated with a higher risk of hospitalization in the older age groups [20]. Nearly 80% of children are exposed to RSV by age two, 100% to hMPV by age five and 90% to hPIV by age five [8]. hPIV are a significant etiology of LRTI in children [21], second only to RSV [11], and AdV are the second most common viral pathogen in children under two years of age [22].

Hospitalized patients with influenza infection have been described in detail in Egypt [23], Jordan [24] and Oman [25]. However, previous studies of other respiratory infections in the region have focused primarily on pediatric cases less than five years of age. Within this age group, the prevalence of RSV in our study population was higher than previous studies of outpatients in Egypt [26, 27] and Oman [28], suggesting possible increased severity for RSV infections, but lower than that identified in studies of hospitalized patients in Jordan conducted in winter when prevalence is expected to be highest [29, 30] and higher than a smaller study in Upper Egypt [31]. AdV and hMPV were found at levels comparable to previous studies of both outpatient and inpatient populations [22, 26, 28, 31]. Studies of hPIV are more difficult to compare since some studies have not specified virus types, but overall prevalence in the current study appears to be similar to previous studies in Egypt [26] and Oman [28].

The seasonality of the viral respiratory pathogens examined in this study generally followed that which has been documented in temperate climates. As in temperate climates, influenza was most common in winter months between October and February [32]. RSV showed circulation throughout most of the year but was highly seasonal, with annual peaks between November and February. This finding is consistent with both temperate North America [8] and with previous studies that have identified RSV seasons in Egypt, Jordan, Qatar, Saudi Arabia and the United Arab Emirates within the same time frame [33] and with the highest peak in January [30]. The height of hMPV circulation was between January and May, which is consistent with its pattern of infection in temperate climates, where peaks occur either with or just after RSV in late winter or spring [7]. For AdV, peaks were noted between April and July, which is comparable to circulation patterns in temperate climates where the virus is seen throughout the year with outbreaks in late winter, spring or early summer [8]. The seasonality of hPIV1 was similar to, though earlier than, virus circulation patterns in the United States [10], and variations in hPIV2 infection were consistent with patterns noted in the United States [10, 11]. hPIV3 follows an annual pattern in the United States, with annual peaks between April and June [10]; a pattern was also noted in the EMR with peaks between May and August in most years.

Although PCR has been established as a valid diagnostic assay with high sensitivity and specificity for respiratory viruses examined in this study [8, 34], the clinical implications of positive laboratory results are less straightforward. The relationship between RSV infection and clinical disease has been established, as infections among asymptomatic individuals are rare [35, 36]. AdV infection levels in asymptomatic children and adults vary [35, 37, 38], though this may be attributable to differences in sampling methodology since throat swabs may detect latent AdV DNA in tonsil tissue [35]. Studies suggest that asymptomatic infection with hMPV is rare among children [39–41], but results from adult populations are less definitive, with reports of varying levels of infection among asymptomatic individuals [36, 42, 43]. Even fewer data are available on hPIV infection in asymptomatic individuals, so the clinical impact of hPIV infection is unclear.

The clinical implications of positive laboratory results are further complicated by co-infections. Multiple viral respiratory pathogens were identified in 501/9,316 (5.4%) samples for which testing for the full panel of viral respiratory pathogens, as well as influenza, was performed. Co-infection with two or more viral respiratory pathogens is common and has been noted in previous studies of viral respiratory infections among pediatric populations in the region [26, 29, 44]. Multiple infections complicate diagnosis because the relative clinical impact of each pathogen is unclear [45], and certain pathogens, such as AdV, the most common co-infecting pathogen identified in this study, are routinely found in the upper airways [46]. There are suggestions that co-infections may increase severity of disease [8, 9], but this conclusion has not been found consistently [4, 46]. The observed impact of co-infections on disease severity may be affected by variations in virus circulation or study methodology [9], and so further studies with consistent testing for a full panel of pathogens are needed to validate the impact of multiple viral respiratory infections on clinical outcomes.

In analyses to assess associations with severe outcomes in this study, there was strong evidence that individuals with SARI and hMPV infection were less likely to experience a severe outcome than individuals with SARI not infected with hMPV, after controlling for age and pre-existing chronic disease. Among participants aged less than five years, there was strong evidence that those with AdV infection were more likely to experience a severe outcome than participants not infected with AdV in this age group. There was also some evidence that influenza infection may be associated with a severe outcome in participants less than five years of age, but a larger study is needed to fully assess this association and associations between other viral respiratory pathogens and severe outcomes. In these analyses, pre-existing chronic disease was the strongest predictor of a severe outcome among participants in this study. Because of associations between chronic disease and age, older participants in this study were at greater risk of severe outcomes, despite low levels of infection with pathogens of interest.

There are several limitations to the current study. Not all enrolled SARI cases were sampled and tested for all viral respiratory pathogens throughout the course of surveillance for a variety of reasons including patient refusal at the time of sample collection, limited sample quantity following influenza testing, problems with sample transport and occasional interruptions in the availability of necessary supplies. Therefore results are limited to a subset of the full spectrum of hospitalized patients with severe respiratory infections and cannot be used for burden estimates. Additionally, samples were collected through routine surveillance activities rather than through random sampling, which further limits the applicability of findings. Because testing focused on a limited panel of viral respiratory pathogens, other viral and bacterial etiologies which may have been factors in co-infections or severity of disease were excluded. The incorporation of testing for additional pathogens or testing for the same pathogens among outpatients or in an asymptomatic control population in the region would offer an opportunity to address some of the challenges of interpreting results from this study. Furthermore, despite monitoring efforts, data completeness, as noted in various denominators in the tables, limited data analyses and affected confidence intervals.

Despite these limitations, the current study provides a substantial amount of information the viral etiology, seasonality and severity of respiratory infections among hospitalized SARI patients in the Eastern Mediterranean Region. As SARI surveillance continues and expands through the EMARIS network, it is important to maintain and improve surveillance for viral respiratory pathogens in order to monitor changes in the etiology, severity and seasonality of pathogens of interest.

## Supporting information

S1 Table Case definitions for severe acute respiratory infection (SARI) used in sentinel surveillance (2008–2014). (PDF)

S1 Dataset (XLSX)
