# Supplementary material for: Viral etiology, seasonality and severity of hospitalized patients with severe acute respiratory infections in the Eastern Mediterranean Region, 2007–2014
Source: PLoS One. 2017 Jul 13;12(7):e0180954. doi: 10.1371/journal.pone.0180954 (PMC5509236; doi:10.1371/journal.pone.0180954)
Supplement: S1 Table — (PDF) [file pone.0180954.s001.pdf]

S1 Table: Case definitions for severe acute respiratory infection (SARI) used in sentinel surveillance (2008-2014)

2007-2009	2010-2011	2012-2014
• Age 2-59 months, AND<br>• Hospitalized, AND<br>• Cough OR tachypnea, AND<br>• At least one sign of pneumonia*<br><br>OR<br><br>• Age ≥ 5 years, AND<br>• Hospitalized, AND<br>• Fever (≥38°C), AND<br>• Cough OR sore throat, AND<br>• Shortness of breath OR difficulty breathing	• Age ≥ 31 days, AND<br>• Hospitalized, AND<br>• History of fever OR current fever (≥38°C) OR current hypothermia (<35.5°C), AND<br>• At least one sign of respiratory infection†<br><br>OR<br><br>• Age ≥ 31 days, AND<br>• Hospitalized, AND<br>• At least one physician assessment criterion‡	• Hospitalized, AND<br>• Fever (≥38°C) within last seven days, AND<br>• Cough<br><br>OR<br><br>• Hospitalized, AND<br>• Clinically-suspected respiratory infection

* Nasal flaring, chest indrawing, inability to breastfeed, vomiting, grunting, convulsions, stridor, tachypnea or lethargy

† Abnormal breath sounds, tachypnea, cough, sputum production, hemoptysis, chest pain, sore throat or dyspnea

‡ Severe influenza-like illness, suspected pandemic H1N1 2009, suspected or x-ray confirmed pneumonia or other respiratory illness
